# Research fellowship in a Lebanese academic medical center: What does it take to shift from an informal to a formal program?

**DOI:** 10.1371/journal.pone.0278576

**Published:** 2022-12-01

**Authors:** Marlene Chakhtoura, Elsa Karam, Elie A. Akl, Rola El Rassi, Gladys Honein-AbouHaidar

**Affiliations:** 1 Clinical Research Institute, Department of Internal Medicine, American University of Beirut Medical Center, Beirut, Lebanon; 2 Hariri School of Nursing, American University of Beirut, Beirut, Lebanon; Duke University Medical Center: Duke University Hospital, UNITED STATES

## Abstract

Research fellowship programs help medical graduates acquire research skills for an academic career. While our institution employed a large number of research fellows, it did not offer them a formal training program. This study aimed to assess the views of fellows and their mentors regarding the current research fellowship program, and to seek their suggestions for a formal training program at our medical center. We conducted a qualitative descriptive study using both focus group discussions and individual interviews with research fellows, and individual interviews with their mentors. We recruited all eligible participants by email. We collected data in person and analyzed it thematically. We followed the consolidated criteria for reporting of qualitative research (COREQ) checklist. A total of 17 research fellows and 17 mentors participated in the study. Participants described the current non-formal program and proposed suggestions for a formal training program. The identification of available vacant positions and the recruitment process followed an unstructured approach, through networking with mentors and previous fellows. Although there is a formal contract, there is no job description, and no definition of roles, responsibilities and rights. Some fellows get the opportunity of being involved in all aspects of research and benefit from a favorable mentor-mentee relationship. Conversely, others struggle with authorship and with the projects allocated to them, some being “non-research” related. Not all fellows end up publishing their projects. Participants provided suggestions to shift into a formal training, including measures to improve on the recruitment process of fellows, defining roles and exposure to all aspects of research. Research fellows are eager to learn, but the currently available program is unstructured. They need a formal training program that meets their expectations, one that offers equitable learning opportunities and benefits to all.

## Introduction

Research fellowship programs provide young medical graduates research training opportunities and prepare them to become independent scientists. In medical disciplines, a research fellow is “a graduate who holds a masters or a doctoral degree in a health science discipline, residency or equivalent clinical experience” [1]. Several models of research fellowship training are currently available in the United States. They typically aim at enhancing fellows’ knowledge and skills in research [2–9], through didactic teaching, work-in-progress sessions [2–9], and close research mentorship [10]. These experiences typically yield to publications and presentation of projects at national and international scientific meetings, as well as opportunities for networking with peers, junior and senior investigators [2–9].

The American University of Beirut-Medical Center (AUB-MC), in Lebanon, has invested in expanding its research programs over the last decade. Scientists from various academic departments recruit research fellows on a yearly basis to assist them in the conduct and the implementation of their research projects. A research fellow usually commits for a full-time one or two years of research with minimal interruptions for travels and interviews for residency positions [2].

As a formal and structured training program for research fellows at our institution was not available, there was a need to design one, providing equitable and optimal learning opportunities to all of them. This study constitutes the first formative phase of a project aiming at establishing a “Research Fellowship Program” at AUB-MC. It encompasses the general and target needs assessment of the Six-step approach in developing, implementing and evaluating curricula for medical education, as described by D. Kerns [11]. We aimed to assess the views of the fellows and their mentors regarding the current research fellowship program, and to seek their suggestions for a formal training program at our medical center.

## Methods

### Study design

This is a qualitative descriptive study, based on focus groups discussions (FGDs) and in-depth interviews with fellows and in-depth interviews with mentors at AUB-MC. Through considering the perspective of both fellows and mentors, this study illuminates our understanding of a comprehensive range of factors of the contextual and the individual factors influencing the fellowship experience.

We received ethical approval from the Institutional Review Board (IRB) at AUB-MC to conduct the study, under the protocol number IM.MC.01. All study participants provided oral informed consent. Written consent was not required, as the risk associated with the current study was below the minimal risk. We audio-recorded the verbal consent of participants at the beginning of the FGD or interview. The Clinical Research Institute (CRI) at AUB-MC provided the logistical support for the study conduct. We followed the COnsolidated criteria for REporting Qualitative research (COREQ) Checklist, as suggested by Tong et al. [12] (See S1 Table: COREQ Checklist [13]).

### Study participants, sampling and recruitment approach

We used the purposive sampling approach in the recruitment of research fellows and mentors at AUB-MC.

#### Research fellows

We invited full time research fellows enrolled at AUB-MC for the two academic years: 2016–2017 and 2017–2018. We obtained the list of research fellows from the human resources department at AUB-MC, which accounted for 66 for the year 2016–2017, and 81 for the year 2017–2018.

We sent an email invitation to all research fellows, followed by two reminders one week apart for those who did not respond. In order to increase the response rate, we used a snowball sampling approach, whereby we asked the fellows who accepted the invitation to invite their peers to participate.

#### Mentors

We sent an email invitation followed by two reminders to 23 mentors at AUB-MC, identified on the Clinical Research Portal Website [14].

### Data collection methods

We conducted FGDs with fellows as well as individual in-depth interviews to accommodate those who were not available at the time of the FGDs. We used individual in-depth interviews with mentors. Our aim was to get the perspective of all eligible fellows, but some did not respond to our invitation so we resolved to those who did. However, we believe that we were able to reach saturation, especially after triangulating their data with those of their mentors.

#### A. Focus groups discussion

We used an interview guide, with probing questions on the responsibilities, the challenges, and the learning experience of the research fellows, as well as their suggestions for a formal research fellowship training program. FGDs took place in a private conference room at our institution. Each FGD was moderated by one of the investigators (EA, MC, GHA) [15], while a fourth investigator (RR) observed all FGDs and took notes. GHA had no prior knowledge with the participants while MC, EA and RR knew some of them but without any established professional relationship. Each FGD lasted around 60 minutes and was audio-recorded. The discussion was mainly in English interjected by some statements in Arabic.

#### B. In-depth interviews

The principal investigator (MC) conducted the in-depth interviews with mentors at AUB-MC. Given her medical profession background and being a faculty at the same institution, she had a prior professional relationship with some mentors. Similar to FGD, the interviewer used the same interview guide that included a number of probing questions. All interviews were conducted in English. The duration of each interview was 30 minutes.

### Data analysis

We transcribed verbatim all FGDs and in-depth interviews. Simultaneously, we translated Arabic statements into English. One investigator conducted the initial coding (EK), with the input of the senior researcher (GHA) on the coding process. The research team met on several occasions to discuss the analysis.

We followed the six phases of the thematic inductive analysis, suggested by Braun and Clarke [16], including familiarization with the content of the transcripts, line-by-line coding of the transcripts, creating a log of candidate themes and sub-themes, refining the final themes, ensuring that the final themes align with the research question and the last step was to interpret the findings and prepare the narrative of the results.

First, the coder familiarized herself with the content of each transcript. She read and re-read each transcript while taking notes on what the data mean. This was followed by a systematic line by line coding capturing the essence of the data. After coding one transcript, she met with the senior researcher (GHA) to ensure congruence in the interpretation of meanings. She then proceeded in coding the remaining transcripts independently. For phase 3, GHA and EK met to discuss the relationship between the codes and to create a log of candidate themes and sub-themes. In phase 4, MC, GHA and EK reviewed those candidate themes and refined them based on a consensus. At this stage, they were mainly concerned to identify the relationship between the themes, both hierarchically and laterally. In phase 5, the research team met to finalize each theme and sub-themes and ensure that the results are congruent to the research question. In phase 6, the narrative of the results, including the research team interpretations of the findings, supported by quotes from interviewees was prepared.

In addition, we triangulated the themes elicited from fellows and mentors to establish similarities and differences, as well as expansion of themes between those two actors. To ensure privacy, we assigned a code to each participant. In the quotes included in the results section, we refer to FGD participants as “FGxPx”, and to interview participants as “Mx” or “Px”, to refer for mentors and fellows, respectively.

### Trustworthiness

To increase credibility, we adopted two strategies. We audio recorded all conversations, transcribed them verbatim, and used them as the main data repository. We used triangulation between two different actors. As for confirmability (i.e. objectivity), we used three strategies. First, we described the study methods in explicit details. Second, two investigators (EK and GHA) met to reach agreement on the coding strategy, and thereafter, on the candidate themes. All team members were involved in the final data analysis, so as to avoid misinterpretation of the results. Third, given that one research team member (MC) had prior relationship with the participants, we used reflexivity, whereby team members were mindful of their personal biases when doing the analysis in order to avoid any subjective interpretation of findings. We used a semi-structured interview guide to increase the dependability of our research. For authenticity, we reported disconfirming results.

## Results

**Fig 1:**
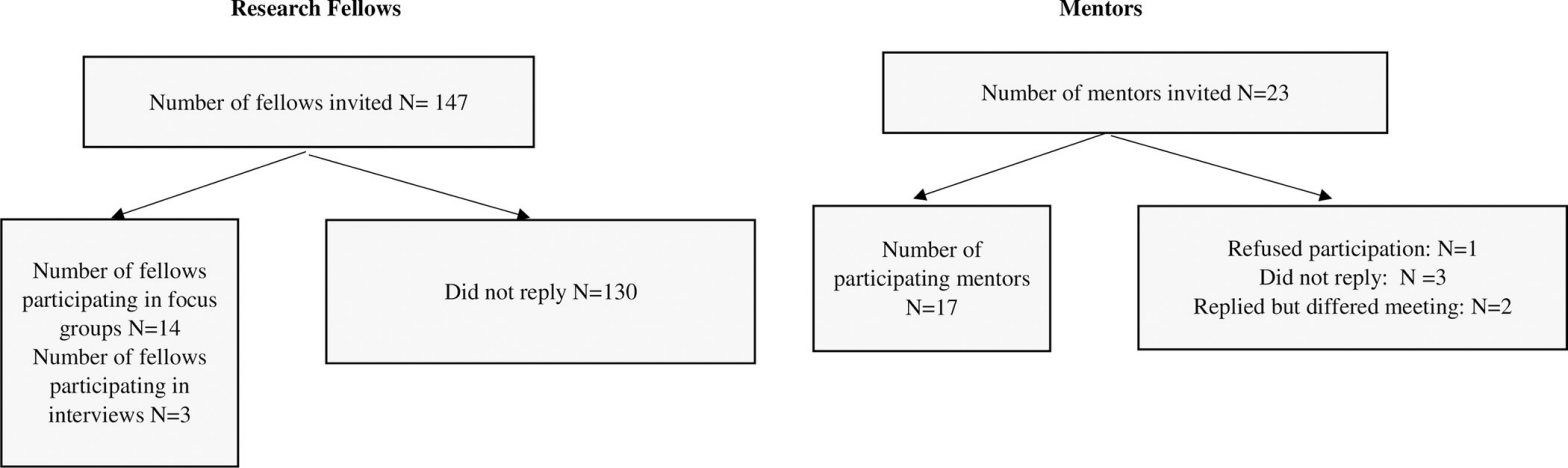
Flow diagram.

We conducted 5 FGDs and 3 interviews with a total of 17 research fellows (10 and 7 participating from the academic years 2016–2017 and 2017–2018, respectively); see flow diagram -Fig 1. All participating fellows were medical doctors recruited for a clinical research fellowship position at AUB-MC, immediately after their graduation from medical school. We interviewed 17 mentors who regularly hire research fellows (see flow diagram -Fig 1).

The inductive thematic analysis led to the following two main findings:

1. Description of the current non-formal training program
2. Proposed suggestions for a formal training program

### 1-Description of the current non-formal training program

Table 1 summarizes the current non-formal training program from motives to recruitment process to outcomes.

**Table 1:** Summary of results on fellows’ and mentors’ perspective for theme 1 that corresponds to the description of the current non-formal training program from motives to recruitment process to outcomes.

Sub-theme	Fellows’ perspective	Mentors’ perspective
**Fellows’ motives: personal and academic**	**Personal** **Buying time to sit for an exam** “*I get some time into some exams” (FG1*.*P3)* **An opportunity when all other options failed** “*to apply for the residency*, *for the match so I’m also—like I knew that I would take one or two gap years” (FG2*.*P1)* **Academic** **Learning more about own specialty** “*You also learn a lot more if you’re interested in a specific field*, *you get greater insight about it*, *you have more time to do a lot of literature reviews and you kind of learn a lot from it as well” (FG1*.*P3)* **Publications** “*Research sounded like a good opportunity to get some papers published*, *maybe enhance the CV” (FG2*.*P2)*	-
**Unstandardized and selective recruitment process**	**Word of mouth and network** “*First of all*, *somebody has to have an interest in the topic*. *Then do—go on doctor-doctor basis to find out if they’re providing any research experience*. *This is usually the connections that you make or by word of mouth; Also*, *networking with previous generations that have worked with researchers before helps in that scenario” (FG1*.*P4)* **AUB graduates are at an advantage** “*The attending already knows about your background*, *about your medical degree” (FG1*.*P4)* *“I noticed it after—after I took the SHARP course*, *a lot of emails started pouring in from attendings” (FG1*.*P5)*	**Word of mouth and network** “*a lot of …[was] word of mouth (M1)* *“Interview is very very crucial*, *and the CV too*, *and the letter of recommendation*, *and it’s better to talk to the people who recommended [her]*, *because most people get shy when they write a letter of recommendation*, *but when you call them on the phone*, *it’s much better*, *they will tell you honestly if they are good or not” (M3)*
**Unspecific terms of engagement**	**Unspecific contract** “*One thing that I think is important is that when I first applied for a research position and I signed my contract I realized that my contract was…… an e-mail practically speaking*, *that said “this PI wants to have you as a research fellow… And I expected a contract to read…”* (*P5)* **Unable to negotiate** “*It wasn’t really negotiated*. *Because I didn’t know that you can actually say that to the attending because you know you’re making me work on this even though it’s not my project*, *if I’m working for several weeks on it”* (*P3)* **Advantages: time flexibility** *“The benefit of this [unspecific contract] is that during this year a lot of us after we apply for a match have to take a long vacations [sic] to go for interviews*, *we need to…” (P4)* **Advantages: room for negotiation** “*One of the things I negotiated with my PI when I first started was—I asked him that I wanted clinical experience in this research position*. *And I wanted to see patients because I knew that was important*.. *he proposed my helping the ED physicians by giving shifts on weekly basis*, *so I did part time research during the week and a shift or two during the week as well*, *and that would be included in my time*. *It’s part of the program but I think it filled a need for both him and for me” (P5)*	-
**Met and unmet expectations**	**Expectations** **Interest in conducting research** *“Would be involved in research from a to z*. *Thinking about your own research*, *planning that out*, *having good guidance*, *writing it up*, *doing the data collection if it’s necessary*, *doing the research beforehand*, *write-up submission*, *review*, *and ultimately the aim is to publish… and*, *to be confident to be doing—to be able to do it by yourself…” (FG1*.*P4)* **Guidance from mentor** *“You need guidance of the mentor*, *they know what’s—they know the field*, *specialty*, *what kind of research is to be made*.. *We go out of med school we barely know anything about [research] what new can be added to the literature etc*..*” (FG2*.*P2)* **Working within specialty** “*When I took this position and some of it included picking up on projects that have already started and starting my own project but it was already clear from the beginning that even if I want to start my own projects they were going to have to be under a certain topic or a subject that the PI is interested in or [pause] works in” (FG1*.*P3)* **Met expectations** **Satisfied with the experience** *“I definitely*, *I did learn a lot about that part of the research*, *like you know*, *the data collection…the field work type*. *I think*, *I did gain a lot of skills*, *especially in terms of like coordinating*, *you know*, *a project at this level*, *you know*, *communication skills*, *definitely I would’ve liked to learn more in terms of—especially data analysis that side I feel—which I could still learn in the coming year hopefully” (FG2*.*P1)* **Benefits working on several projects** *“Which was a good contrast because I got the best of both worlds in a sense where*, *you know*, *whether I worked on a huge project that needed years of research and then I also worked on short term projects which I could call my own” (FG1*.*P4)* **Mentor-mentee relationship satisfaction** “*When it comes to research*, *she [PI] knows everything*.. *she knows how to direct you and if there’s any—we have questions*, *she knows the whole like—what’s being done everywhere… so she can tell you “oh they’re doing this*, *that thing” or that kind of thing*. *Yeah*, *so she’s very helpful*, *she’ll sometimes send like articles like we should think about doing this*, *and like we just came—she came back from a conference and she said “this is what they’re doing*, *maybe we could do something like that” (P2)* *“We have weekly meetings that are set every week*, *we’re communicating like every day via e-mail or phone or whatever*. *On the contrary*, *we are on the same page about what’s going on which is great*. *Sometimes there’s a bit of micromanaging on his [PI] part but I guess each one has his own style*.* *.* *.*” (FG2*.*P1)* **Teamwork** *“I think research is like a ladder of process*, *you need to help others and you need others to help you*, *you can’t do it all by yourself*, *so yeah” (P2)* **Unmet expectations** **Process of allocation to projects and large number of projects** “*I would prefer*, *like she said*, *to work on two or three projects rather than on many projects” (FG1*.*P5)* *“I started many different projects*, *maybe ten if not fifteen like fifteen different projects and at different points in time I found out that this’s probably not feasible*, *not doable and out of trial and error I learned what was good and what was not*. *I didn’t expect that” (FG1*.*P4)* **Unable to do own project** “*At the end*, *you don’t feel you achieved something*, *even though you did put a lot of work on the project*, *but you feel like at the end*, *you didn’t get any credit basically*, *or you know*, *that was the main thing that was frustrating for me*, *in terms of productivity and outcomes what do I have to show for myself at the end of the year” (P1)* **Working on unrelated research tasks** *“I had to do it once and then the second time honestly I*, *I kinda [sic] refused because I didn’t feel that I could—it’s for example screenshotting a lecture from a video or—to prepare something (FG1*.*P2); It’s like a secretary*, *“book a ticket” or something” (FG1*.*P2)*, or *“proctor for exams for just within like the department so they feel like they were personal assistants so” (FG1*.*P5)* **Transparency in authorship allocation** “*Authorship tends to change a lot which isn’t something that should be happening but like towards the end when you’re*, *you know*, *when you’re almost done with the project you start to see like—the order starts changing based on what the PI thinks” (FG1*.*P5)* **Overall disappointment** *“A lot of time consuming projects and [pause] no credit*, *no recognition*, *and this is something that—not about being not recognized but something that I don’t think I should’ve been involved in from the beginning*, *you know” (FG1*.*P3)* **Mentoring** **Not enough time with the mentor** “*more communication*.. *more time—more one on one time*. *I think the whole year*, *we had like seven hours of—or seven days let’s say of one on one time*.. *Otherwise all by e-mail communication” (FG2*.*P2)* **Hierarchical relationship** The workload is heavy *“The attending would underestimate how much time a specific task*, *a certain task would take and then so for example I wanna work on a specific project and I think it’s the most valuable one and it’s the most time consuming*. *And then they would keep on sending me other tasks to do*, *for example review another—a paper that wasn’t published in the past or a few other tasks” (FG1*.*P2)* **Organizational** **Delayed contracts, delayed email account and payments** “*I was first paid in the middle of the third month” (FG2*.*P2)* *“It was annoying because you don’t get access*, *to privileges that you don’t get…we have to like call IT to get temporary accounts just to be able to log in*.. *I remember the first month I was using the account of my predecessor*, *and there was a bit of a mess*, *because sometimes—at some point I even lost some data because his account expired and mine hasn’t started so had to get IT to luckily recover the data*. *It was a bit of a mess the first few months finally I got my own account*. *So that transition period was a bit…” (FG2*.*P1)* **Limited funding to hire assistant staff** **Lack of training on softwares and administrative tasks**	**Expectations** **Interest in conducting research** **Fellows to be available** **Applying ethical considerations** **Unmet expectations** **Some fellows’ primary motive was not research** “*Most of them are doing it so they don’t waste a year*, *at the same time they want to prepare for their steps and they want to strengthen their CV” (M5)* **Availability** “*I think the main challenge would be the availability time” (M4)* **Residency interrupted their fellowship** “*The biggest problem is that they’re—already it takes them so long to get oriented and caught up and then they go for an interview for a month in the middle of it*, *and then they need to wrap up and leave early because they’ve matched somewhere” (M1)* **Mentoring** **Clinical workload** “*Clinicians*, *when we kind of work for our income*, *so we don’t really have time dedicated to research*, *set up big projects” (M6)* **Not enough experience in leading research project** **Not passionate about research, did it for promotion’s sake** **Organizational** **Little coordination between departments** **Lack of funding strongly impacted research fellows’ recruitment, and other research staff**
**Research Fellowship Outcomes**	**Hands-on experience on research** “*The more I talk to research fellows*, *I am discovering new things*..*—For example*, *I use Endnote*, *other people use other referencing frameworks…it wasn’t just referencing*, *when it comes to analysis*, *some people like use books or—just to freshen up on the material” (FG1*.*P5)* **Networking** “*Also I really appreciate*.. *Whenever I write a paper*, *I send it to friends to reread it*, *just for basic review*.* *.* *. *Making connections with other research fellows*, *sometimes you can just find somebody else just to read your paper just for medical comparison” (FG1*.*P4)* **Publications**	**Large variability in productivity depending on individual fellow** “*And so the productivity of the research fellow has been quite variable depending on how aggressive they are and how eager they are to*, *you know*, *participate in more than one project and succeed in finishing the projects” (M 7)*

Abbreviations: AUB: American University of Beirut; ED: Emergency department; IT: Information technology; SHARP: Scholars in Health Research

#### A. Fellows’ motives

Fellows reported that the research fellowship experience offered them better chances in getting accepted into “… *a better residency program (FG2*.*P2)*” in the United States as *“research experience is a must (FG1*.*P4)”*. For those preparing for their United States Medical Licensing Examination (USMLE) exams, the fellowship provided them with time flexibility to prepare for their tests, and later on, for their residency interviews. The fellowship also provided a needed financial support. For a few, research fellowship filled a gap when all the other options failed. Many others pointed to academic motives, including interest verging towards being “*passionate about research (FG1*.*P5)”*, acquiring in-depth knowledge on a specific medical specialty or sub-specialty, and an opportunity to get more publications. For some, the research fellowship year helped them choose their career path, i.e. academic or clinical track, or even public health.

#### B. Unstandardized and selective recruitment process

Both fellows and mentors indicated no repository for vacant positions and that the process of hiring was not standardized. Few mentors advertised their vacant research positions, but “*a lot of … [was] word of mouth (M1)*, which was confirmed by fellows, who indicated that they depended on their network and direct communication with mentors or previous fellows to identify available opportunities.

Fellows added that graduates from the same medical school (AUB) were at advantage for two reasons: first, they know most of the mentors; second, some AUB graduates had completed a research certificate, such as the Scholars in Health Research Program (SHARP) [17], which enhanced their chances to be offered a research fellowship position *“I noticed it after—after I took the SHARP course*, *a lot of emails started pouring in from attendings (FG1*.*P5)”*.

Some fellows indicated that the available positions were not always timely, hence they were engaged in positions not matching their interest. “*I was working with the PI before—in Med school…so I sent out an e-mail saying “I wanna work with a person in neurology and such”*, *and so when I was finishing up my project and I knew I was going to graduate*, *I asked if there was a position open for me*, *and then she told me “yes*, *just apply” and I applied (P1)”*.

Then, mentors interviewed the candidates and selected the best match. Some mentors targeted those who were interested in spending more than one year in research and those who were not sitting for the USMLE exams during their research year.

#### C. Unspecified terms of engagement

Both mentors and fellows indicated that research fellowship positions did not have a clear job description. The responsibilities and the roles assigned to the fellow were left utterly to the mentor’s decision and the available resources. Mentor and fellow met upon the fellow recruitment, to discuss mutual expectations. Typically, there was no orientation or training, but rather previous fellows handing over their projects to the newcomers. “*I had talked during the handover about all the projects that were running which were like three at the time*. *I kind of knew what was expected*. *But I didn’t know exactly what my role would be*… *(FG2*. *P1)”*.

Fellowship opportunities were not always funded by research grants. Therefore, some fellows were paid, while others worked as volunteers. Paid fellows usually signed a contract with the human resources department. However, this contract did not stipulate the working hours, vacations, rights nor duties. Most of the fellows indicated limited opportunities to negotiate their contract with the mentor. Interestingly, the unspecific contract allowed more time flexibility to sit for USMLE exams and leave intermittently for their residency interviews in the United States. In addition, it provided room to later negotiate their role with the mentor. Volunteer research fellows did not have to sign any contract, did not get any job-related benefits, and sometimes not even authorship on projects they worked on.

#### D. Met and unmet expectations

*Fellows’ perspective*. Upon enrollment, fellows expected to be involved in all research aspects, and to enjoy some independence, while receiving the appropriate guidance from mentors. They were also aware that their work would be limited to research projects of their mentors, and thus unable to conduct their own projects.

Several fellows were “*satisfied*… [and had] *“the experience* [they] *wanted” (FG2*.*P2)*. The friendly work environment and the teamwork allowed for a smooth transition and hand over of various project, from one fellow to another. Indeed, the availability of other research team members, such as a research assistant or a coordinator in some units, helped the fellow in both research and administrative tasks. For some, working on several projects was beneficial. Some fellows described their mentor-mentee relationship as “*good*”, the mentor being supportive and always generating ideas for new projects, and others learned teamwork and how to appropriately manage their time.

Conversely, some fellows expressed an overall disappointment *“We*, *unfortunately*, *are—they think our lives are dedicated to this research*, *throughout the day*, *twenty-four seven*, *it’s not how it works (FG1*.*P1)”*.

Many reported unfair project allocation, leading to working on a large number of projects, sometimes unrelated, or doing someone else’s assignments. Consequently, they were not able to complete their projects. Some were even assigned tasks that are not related to research, such as administrative tasks, *“Preparing PowerPoints to a conference over the weekends*, *they send you an e-mail on Saturday night*, *and you end up working all day tomorrow* (*P1)”*. They would accept those tasks in order to get a good recommendation letter, the only documentation that they have successfully completed their fellowship.

Fellows also complained that mentors were not available to provide guidance or career advice. “*I feel that my PI is very busy*. *I barely have time to sit down and discuss the projects (FG1*.*P3)”*.

At the institutional level, fellows described delays in contracts leading to delays in payment, and in privileges (e.g., access to email). There was a lack of training on softwares (such as Oracle), crucial for their daily research related financial activities, resulting sometimes in delays in grants administration.

*Mentors’ perspectives*. Mentors expected their fellows to be interested in academia, to know research ethics regulations and to be dedicated for their work. They expected them to at least to have the basic research skills (i.e.in biostatistics, literature search, writing skills). Mentors pointed to many unmet expectations from their fellows: primary motive not being research, not being available all the time (due to studying for exams and traveling for residency interviews), and lacking some skills (e.g., scientific writing). Mentors also complained that some fellows might have opted for research only because they did not have any other option. They also noted that the one-year commitment is very short considering the time needed to get oriented, and the time spent on residency interviews. Mentors also described their own failure in meeting fellows’ expectations. They indicated that their clinical workload gave them little time to closely oversee their fellow’s work. Others candidly admitted lacking experience and skills to lead research projects. Some admitted they were not passionate about research, and they only did it for promotion’s, with little coordination between the relevant departments (e.g., IRB and the Office of Grants and Contracts). Also, the lack of funding strongly impacted the ability to recruit research fellows and other research staff, such as a research nurse or a research unit coordinator, to assist both mentors and fellows.

#### E. Outcomes of the research fellowship

*Fellows’ perspectives*. For most fellows, the research experience was fulfilling. They reported acquiring hands-on experiences on how to select a successful project, collect and analyze data, conduct a trial, build a database, and perform a literature search. They also said they learned how to establish a network of peers, exchange ideas, share challenges and seek advice on problematic situations. Yet, the ultimate outcome was publications, which was not met all times.

*Mentors’ perspectives*. Mentors agreed that the research fellows’ productivity was “*variable*”, depending on their interest in research, their motivation and their dedication. Some were hard workers and were able to publish four or five papers in one year, while others did not publish any.

### 2-Proposed suggestions for a formal research training program

Table 2 provides a summary of suggestions for the structure and content of the proposed formal research program.

**Table 2:** Suggestions for research fellowship and suggestions for a formal program.

Sub-theme	Fellows perspective	Mentors perspective
**Structure**	**Clear definition of roles (contract)** *“Yes*, *I do suggest to have a definition of roles at the beginning of the contract” (FG5*.*P1)* **Database matching interest in position** *“If there would be a way to have—be more*, *like have a more systematic or standardized way of announcing vacancies*, *I think that would make everybody’s lives easier” (FG2*.*P1)* **Structured time to meet with mentors** *“So you would suggest more like—to dedicate at least an hour every week or some sort where you can just be…” (FG4*.*P2)* **Availability of a research coordinator** *“The availability of a research coordinator and supportive resources would come hand in hand*, *but of course*, *it’s very important because then you have less workload and you focus on the actual research and don’t have to deal with the coordination and the administrative research” (FG5*.*P2)* **Institutional support** *We should include how to deal with databases*, *transfer of database*, *upkeep*. *And for some of the resources also*, *Q-link and other programs that you might use…(FG1*.*P4)*: *So data gathering*, *how to get files from the—patient files*, *where to go to get patient files to go over your patient’s files*. *Q-link program to download some information from*, *you know*, *patient files that are electronic” (FG1*.*P4)* **Getting a certificate at the end of the year** *“A certificate by itself is gonna attract people*, *just because some—to be blunt*, *we’re interested in research and we took this year knowing that we wanna do something with it” (FG1*.*P4)*	**Database matching interest in position**“*So you pair—so if it’s someone who’s an EM graduate*, *they’re paired with the EM faculty*, *someone who’s internal medicine*, *they’re paired with—or maybe*, *it doesn’t have to be necessarily having the specialty but wanting to go into the specialty or an interest in it maybe (M 1)”***A 2-year program***“It needs to come with a commitment of at least two year—two years from the fellows ‘cause I’m not willing to also have my research fellows come and spend so much time getting developed and then for me*, *at the end of the nine months*, *for them to say “I’m leaving” (M1)***SHARP***“I think that if there is a mechanism that they all go through a SHARP type of program then when they read*, *they have some additional things that they can—that can really be valuable for them because take some individual who comes in and works very hard on*, *let’s say*, *a very focused study in bariatric surgery*, *and that individual is interested in cardiac surgery” (M3)***Getting a certificate at the end of the year (quote)***“it would be nice to have an eventual goal of having a certificate for that” (M 2)*
**Content**	**Pre-enrollment orientation and intensive training covering various topics** *“So just like you have*, *in any job*, *probational [sic] period of three months where you’re still taking courses and training*, *this could be part of the training for research fellows to begin with” (FG1*.*P4)* **Incorporate research in residency** “*I also was thinking about*: *in the States they incorporate research into residency programs which would mix research and residency programs and I think this would be a new credible opportunity” (FG1-P4)*	**Pre-enrollment orientation and intensive training covering various topics** *“They have to fulfill certain requirements that are asked from them from the program including certain lectures or workshops or seminars or videos to watch or in-service training*, *requirements in terms of how to conduct certain types of research*, *ethical research*, *if there are animal studies*, *if they need to know about animal and not just human subjects*, *regulatory problems*, *basic common language or updates and this has to be an introductory period at the time when they join*. *So people join at a certain time in the year so that it can be part of an orientation period before they start” (M 4)* **Yearlong program including** “*In our department right now*, *we’re trying to get some basics for them*, *meaning we’re trying to have week—monthly seminar where they present*, *we critique their work*, *we evaluate—we evaluate what they’re doing*, *we give them advice on where they’re going with their projects” (M 5)*

Abbreviations: SHARP: Scholars in Health Research Program

#### A. Structure

Fellows suggested having a “*real* (*FG1*.*P4*)” contract, with a clear definition of the fellow’s roles, responsibilities, and expectations by both parties. Fellows and mentors asked for a database for matching fellows with mentors, based on their interest. *“I wish that there was kind of like a platform where we know who are the attendings and what positions do they have (FG1*.*P5)”*. They requested a structured time allocated to meet with their mentors on a weekly basis to discuss projects, progress, and challenges.

The fellows recognized that the availability of a research coordinator in the unit was extremely helpful in taking over the administrative work, and in coordinating various tasks between research team members. More institutional support was also recommended in terms of availability and training on software for statistics, referencing and others, in addition to resources/guides for application to the IRB, data transfer… “*How to deal with the sensitive data*, *for example*, *if you’re working with data that’s*, *I don’t know*, *the names are anonymous or whether you have access to it*, *something like that*, *you know (FG1*.*P3)”*. Fellows and mentors suggested to make the research fellowship become “*legitimate* (*FG1*.*P4*)”, meaning that it should be a program with a certificate of completion at the end of the year. One of the mentors suggested to have a 2-year fellowship program and another proposed to have the SHARP summer course as the core curriculum for the research fellowship, to be coupled with mentoring by a faculty of a specific specialty.

#### B. Content

Regarding the formal training program, fellows suggested a pre-enrollment orientation to help understand what the field is all about. Both actors indicated the need for more intensive training to be held in the morning or at lunch break, on a weekly or biweekly basis, covering various topics. In addition, fellows suggested to have sessions with experienced mentors, who would help them delineate expectations and provide them with guidance on how to manage their projects. On the other hand, they suggested that some mentors’ skills and competencies need improvement. Few mentors proposed having yearlong sessions dedicated to journal clubs and presentations of the fellows’ project. The mentors noted that the program should not target only fellows in a paid position, but also those volunteering. Some mentors highlighted the importance of having technical resources, from the CRI, for instance, to support research fellows in areas where the mentor does not have the time to do so. “*That fellow can work with the PI but at the same time can utilize resources through that fellowship program (M6)”*. One mentor highlighted the importance of the program as a platform for fellows to establish networks with their peers, and to get the sense of belonging to a certain community “*But it’s a sense of belonging*, *the group together*, *and people discussing and learning*, *this is what I think is the most important (M2)”*. Another one suggested to incorporate research training with residency programs *“I also was thinking about*, *in the States*, *they incorporate research into residency programs which would mix research and residency programs and I think this would be a new credible opportunity (FG1-P4)”*.

## Discussion

This study constitutes the formative phase of a large project aiming at establishing a formal research fellowship program at AUB-MC, and provides a baseline description to build upon. The current research fellowship opportunities have several challenges related to the availability of vacant positions, the unstructured recruitment process, and the ill-defined roles, responsibilities, and rights of fellows.

Although our sample is limited to AUB-MC, we believe that the suggested formal program can be scaled to programs in Lebanon and other low to middle income countries. In a country/region where clinical research is still limited, establishing a strong research training program is meant to support career development of individual fellows and fostering a research environment to build a strong academic workforce. Indeed, a structured research training program enhances career development in clinical research, as it has been shown in various specialties including dentistry [18], surgery [19, 20], pathology [21], family medicine [22] and pharmacy [23]. Such programs have demonstrated an important impact of research training on determinants of academic career, including number of publications, h-index, holding a faculty appointment in academic institutions and receiving competitive funding from the National Institute of Health (NIH) to conduct research projects [18, 20].

At our institution, undoubtedly, both fellows and mentors show motivation and interest in research. Several restraints, related to personal and organizational factors, weaken their engagement. However, they provided suggestions to address those restraints. We took these suggestions into consideration to design a structured research fellowship training program, similar to other programs described in the United States [2–10]. The program constitutes a training opportunity for postgraduate, combining didactic sessions, work in progress presentation, journal club and workshops in advanced methodology, spread out over one academic year. While the sessions have been traditionally face to face, during the COVD-19 crisis, the program continued its various activities through virtual meetings. Upon the completion of the program, fellows receive a certificate of completion.

Challenges in the mentor-mentee relationship reported in this study included lack of time, limited mentorship skills [24], the relationship “power imbalance” and mentors’ authority [25]. There is no doubt that mentors play a critical and influential role in research fellowship outcome and career development [26], and this relationship needs to be addressed in the formal program. Our participants suggested compensation and recognition of faculty working with research fellows, and training them on mentoring, as measures to improve the mentor-mentee relationship. Some fellows recommended improving the mentors’ knowledge and skills in research. Currently, the CRI at AUB-MC is providing research training for young faculty at AUB-MC, under the Faculty Advancement Program, to support them in the conduct their own research projects [27].

Fellows identified challenges related to financial constraints and sometimes lack of unit coordinators and infrastructure to support capacity building and research fellows’ trainings; such challenges were described during the experience in capacity building in South Asia [28] and other low to middle income countries [29]. The availability of unit coordinators to facilitate fellows’ work is partially addressed. Only few programs currently have a research coordinator. The request for more institutional support is currently being undertaken by the CRI at AUB-MC, which provides, through various programs and units, the needed support and education/training opportunities for clinical and translational research [30].

The current process of position identification based on networking with peers and faculty, rather than a structured database is a main limitation. Both fellows and mentors recommended having a database with matching of fellows’ interest with available positions. This possibility needs exploration for feasibility, by the human’s resources department at the level of the institution.

The fellows reported on challenges related to authorship, and this has been previously described in academia. For instance, in one study on medical students involved in research, 26% reported that expectations and criteria for authorship were not clarified [31]. Thus, in our program, we need to ensure that the authorship guidelines are clearly started before engagement.

There is no “formal” contract or specific job description for research positions. Fellows recommended having a formal contract describing their roles, responsibilities and rights. While such a problem may seem to be related to the recruitment and the hiring process at AUB-MC, mentors and fellows are expected to meet and discuss mutual roles, responsibilities and rights.

An important issue that was not highlighted by fellows nor mentors was the training on balancing personal factors and soft skills that is crucial for advancement of physician-scientist [32]. Indeed, leadership, communication and negotiation skills, professional and other soft skills are the pillars of excellence in clinical care [31], while they are often missing from traditional training programs [32].

Several metrics to measure the benefits of clinical research training have been identified and included research productivity, the traditional tool for the evaluation of research training outcomes [33], education, mentorship, promotion of collaboration, networking, work-life balance [34], in addition to improvement in research environment at both the institutional and the national level [29]. Some of these parameters were highlighted by fellows and mentors from our institution as perceived benefits. Indeed, research training impacts physician’s career track, by increasing fellows’ research productivity and helping them build a solid research network. Ultimately, it provides the skills to become an independent scientist and advance steadily in career ladder [35, 36].

## Conclusion

Research training has a major impact on physicians’ academic career. This study identified the views and gaps in the training of medical graduates, specifically related to the identification of research positions, the job description, the expectations of fellows and mentors, and the outcomes of the training program. These findings serve to establish a formal research training program, a prototype to follow in Lebanon and in the region. Several challenges were highlighted; Some of them may be addressed by building a portal for available research fellowship positions, accessible by both fellows and mentors, and by designing a structured program, that ensures equitable learning opportunities and benefits to all fellows. Further investigations are needed to explore the long term impact of such a program in low to middle income countries on fellows’ academic achievement, including publications and successful grants submissions. Finally, additional research is needed to assess the impact of the online research training on fellows’ performance, as this opens the door for fellows from various countries to benefit remotely from research fellowship programs.

## Supporting information

S1 Table The consolidated criteria for reporting of qualitative research (COREQ) checklist. (DOCX)
