# Supplementary material for: Research fellowship in a Lebanese academic medical center: What does it take to shift from an informal to a formal program?
Source: PLoS One. 2022 Dec 1;17(12):e0278576. doi: 10.1371/journal.pone.0278576 (PMC9714842; doi:10.1371/journal.pone.0278576)
Supplement: S1 Table — (DOCX) [file pone.0278576.s001.docx]

**Supplementary Table 1. The consolidated criteria for reporting of qualitative research (COREQ) checklist**

| **Item** | **Description** | **Answer** |
| --- | --- | --- |
| **Domain 1: Research team and reflexivity** |  |  |
| *Personal Characteristics* |  |  |
| 1. Interviewer/facilitator | Which author/s conducted the interview or focus group? | GHAH, EA, MC |
| 2. Credentials | What were the researcher’s credentials? E.g. PhD, MD | GHAH: PhD, MPH, RN  EA: MD, MPH, PhD  MC: MD, MSc |
| 3. Occupation | What was their occupation at the time of the study? | Medical doctors |
| 4. Gender | Was the researcher male or female? | Both |
| 5. Experience and training | What experience or training did the researcher have? | GHAH …  MC has an MS degree in clinical research |
| *Relationship with participants* |  |  |
| 6. Relationship established | Was a relationship established prior to study commencement? | No |
| 7. Participant knowledge of the interviewer | What did the participants know about the researcher? e.g. personal goals, reasons for doing the research | Participants were informed by the researchers about the study aims |
| 8. Interviewer characteristics | What characteristics were reported about the interviewer/facilitator? e.g. Bias, assumptions, reasons and interests in the research topic | The researchers wanted to assess the views, perceptions and challenges of both fellows and mentors, assess their needs to establish a formal program for training of research fellows |
| **Domain 2: Study design** |  |  |
| *Theoretical framework* |  |  |
| 9. Methodological orientation and Theory | What methodological orientation was stated to underpin the study? e.g. grounded theory, discourse analysis, ethnography, phenomenology, content analysis | Content analysis |
| *Participant selection* |  |  |
| 10. Sampling | How were participants selected? e.g. purposive, convenience, consecutive, snowball | Purposive sampling and snowball was used for the recruitment of fellows  Purposive sampling was used for the recruitment of mentors |
| 11. Method of approach | How were participants approached? e.g. face-to-face, telephone, mail, email | Email |
| 12. Sample size | How many participants were in the study? | 17 fellows and 16 mentors were recruited |
| 13. Non-participation | How many people refused to participate or dropped out? Reasons? | We contacted 147 fellows, 17 accepted to participate  All mentors, except one, participated in the study |
| *Setting* |  |  |
| 14. Setting of data collection | Where was the data collected? e.g. home, clinic, workplace | Workplace |
| 15. Presence of non-participants | Was anyone else present besides the participants and researchers? | No |
| 16. Description of sample | What are the important characteristics of the sample? e.g. demographic data, date | Participants were recruited from 2 academic years. We did not collect data on their demographics |
| *Data collection* |  |  |
| 17. Interview guide | Were questions, prompts, guides provided by the authors? Was it pilot tested? | Prompts and guides were provided by the authors. They were not pilot tested. |
| 18. Repeat interviews | Were repeat interviews carried out? If yes, how many? | Interviews were not repeated |
| 19. Audio/visual recording | Did the research use audio or visual recording to collect the data? | Audio recording was used to collect data |
| 20. Field notes | Were field notes made during and/or after the interview or focus group? | Yes by RR |
| 21. Duration | What was the duration of the interviews or focus group? | 60-90 minutes for focus groups  15-30 minutes for interviews |
| 22. Data saturation | Was data saturation discussed? | Yes “we believe that we were able to reach saturation, especially after triangulating their data with those of their mentors |
| 23. Transcripts returned | Were transcripts returned to participants for comment and/or correction? | No transcripts were not returned |
| **Domain 3: analysis and findings** |  |  |
| *Data analysis* |  |  |
| 24. Number of data coders | How many data coders coded the data? | 2 |
| 25. Description of the coding tree | Did authors provide a description of the coding tree? | Yes described as part of the data analysis |
| 26. Derivation of themes | Were themes identified in advance or derived from the data? | Themes were derived from the data |
| 27. Software | What software, if applicable, was used to manage the data? | No software was used |
| 28. Participant checking | Did participants provide feedback on the findings? | Participants did not provide feedback on the findings |
| *Reporting* |  |  |
| 29. Quotations presented | Were participant quotations presented to illustrate the themes / findings? Was each quotation identified? e.g. participant number | Yes in narrative and tables 1 and 2 |
| 30. Data and findings consistent | Was there consistency between the data presented and the findings? | Yes |
| 31. Clarity of major themes | Were major themes clearly presented in the findings? | Yes in tables 1 and 2 |
| 32. Clarity of minor themes | Is there a description of diverse cases or discussion of minor themes? | Yes in narrative and tables 1 and 2 |
